# Complement the cell death

**DOI:** 10.1038/cddis.2016.369

**Published:** 2016-11-10

**Authors:** Joo Guan Yeo, Jingyao Leong, Jinhua Lu

**Affiliations:** 1 Department of Microbiology and Immunology, Immunology Programme, Yong Loo Lin School of Medicine, National University of Singapore, Singapore, Singapore; 2 Division of Medicine, KK Women's and Children's Hospital, Singapore, Singapore; 3 SingHealth Translational Immunology and Inflammation Center, Singapore Health Services, Singapore, Singapore

Different forms of cell death can result in divergent outcomes in tissue homeostasis and immune responses.^1^ Cells that undergo apoptosis are immunosuppressive and display phagocytic signals that culminates to their rapid and silent disposal that is devoid of inflammatory tissue injuries or autoimmunity.^2^ Mechanistic defects in either apoptosis or clearance can render these dying cells inflammatory and self-immunogenic.^3^ In the domain of dead cells clearance, the predominant focus has been on the myriad of ‘eat-me' signals that are presented on dying cells and the phagocytic receptors that mediate efferocytosis. Here we aim to highlight the extracellular proteolytic processes that can complement this clearance mechanism and provide an additional layer of protection against auto-inflammation.

Systemic lupus erythematosus (SLE) is an archetypal autoimmune disease marked by autoantibodies reactive to nuclear antigens (DNA and proteins).^4^ Pathogenically, how these autoantibodies are induced remains ill-defined. Exogenous injection of apoptotic cells in mice, especially in the presence of adjuvant, promotes the formation of autoantibodies. This reveals the intrinsic immunogenicity of dying cells that is often masked during apoptosis.^5^ Cells contain intracellular adjuvant such as IL-1*α*, IL-33 and high mobility group box 1 (HMGB1), collectively known as danger-associated molecular patterns (DAMPs) or alarmins.^6^ IL-1*α*, IL-33 and HMGB1 all serve dual roles as chromatin binding proteins and cytokines. With respect to IL-1*α*, retention in nucleus occurs during apoptosis, as opposed to uninhibited release by necrotic cells, which can incite an inflammatory response.^7^ Likewise, HMGB1, a DNA-binding protein found within the nucleus is released as a chromatin complex during secondary necrosis and these HMGB1-bound chromatins trigger the formation of antinuclear autoantibodies and lupus-like disease in mice.^8^ Of greater mechanistic and clinical importance is the critical role of HMGB1, found in circulating lupus DNA-immune complexes, in inducing the formation of anti-dsDNA autoantibody and the strong positive correlation between the HMGB1 content in these immune complexes and the anti-dsDNA titer in SLE patients.^9^ How other alarmins in apoptotic cells are concealed from activating phagocytes is less clear.^6^

Apoptosis constitutes a series of caspase activation that are involved in the regulated cleavage of a multitude of cellular proteins.^10^ Defect or inhibition of caspases can transform immunosuppressive apoptosis to proinflammatory necrosis. Other enzymes may also be activated during apoptosis that contribute to the dismantling of cellular structures. Concomitantly, intracellular autoantigens and alarmins may be inactivated by these enzymes. Notably, nuclear antigens reactive to SLE autoantibodies were found degraded during apoptosis.^11^ Mechanisms also appear to exist that safeguard against inflammation or autoimmunity when apoptosis progresses aberrantly into necrosis. For example, dying cells release DNA antigens that induce anti-DNA autoantibodies, but these DNA antigens are susceptible to extracellular degradation by DNases, for example, DNase 1 and DNase1L3.^12^

In a recent study, we found that extracellular HMGB1 was also susceptible to protease degradation.^13^ HMGB1 acts as an adjuvant in lipopolysaccharide (LPS) activation of monocytes, macrophages and dendritic cells. It enables low doses of LPS, which is otherwise poorly stimulatory, to activate these cells to produce IL-6 and TNF*α*. HMGB1 contains two cleavage sites for the complement protease C1s and, after C1s cleavage, this adjuvant activity of HMGB1 is diminished as it no longer synergizes with LPS in IL-6 and TNF*α* induction.^13^ This suggests an extracellular mechanism that contributes to the trimming of cellular residues and inactivation of alarmins and probably autoantigens as well (Figure 1).

When dying cells become necrotic and release cellular structures, they also become permeable to extracellular enzymes. In another recent study, we found that, in early apoptotic cells, the complement protein C1q only bound to the periphery and was largely excluded from the nucleus.^14^ In late apoptotic cells, it penetrated the nucleus and bound to the highly immunogenic nucleolus.^14^ C1q, C1r and C1s naturally exists as C1 (C1qC1r_2_C1s_2_) complex and the binding of this complex to the nucleoli activates the proteases C1r/C1s, resulting in the cleavage of numerous nucleolar proteins. Among these C1r/C1s-cleaved proteins are two abundant nucleolar autoantigens, that is, nucleolin and nucleophosmin-1.^14^

Genetic deficiency of any component of C1 complex causes antinuclear autoantibodies formation and are among the strongest risk factors for SLE.^15^ With respect to the implied C1q protection against SLE pathogenesis, the prevailing explanation has been based on C1q opsonization of dead cells for phagocytic clearance.^3^ Impaired clearance contributes to the accumulation of dead cells and the activation of immune cells. Besides swift efferocytosis, growing recent studies stress the importance of an additional arm in dead cell clearance, which we term as `dead cell trimming'.^14^ Both intrinsic and extrinsic degradation mechanisms can contribute to the trimming of cellular structures in a dead cell, for example, proteases (caspases, C1s and so on) and nucleases (Trex1, DNases 1, DNase1L3 and so on), so as to inactivate alarmins and autoantigens and globally dampen the immunogenicity of dead cells (Figure 1).

In conclusion, the orchestration of dead cells disposal is not limited to just physical clearance by efferocytosis but includes the action of both intracellular and extracellular proteases/nuclease acting in tandem to disarm their potential immunogenic effects.

## Figures and Tables

**Figure 1:**
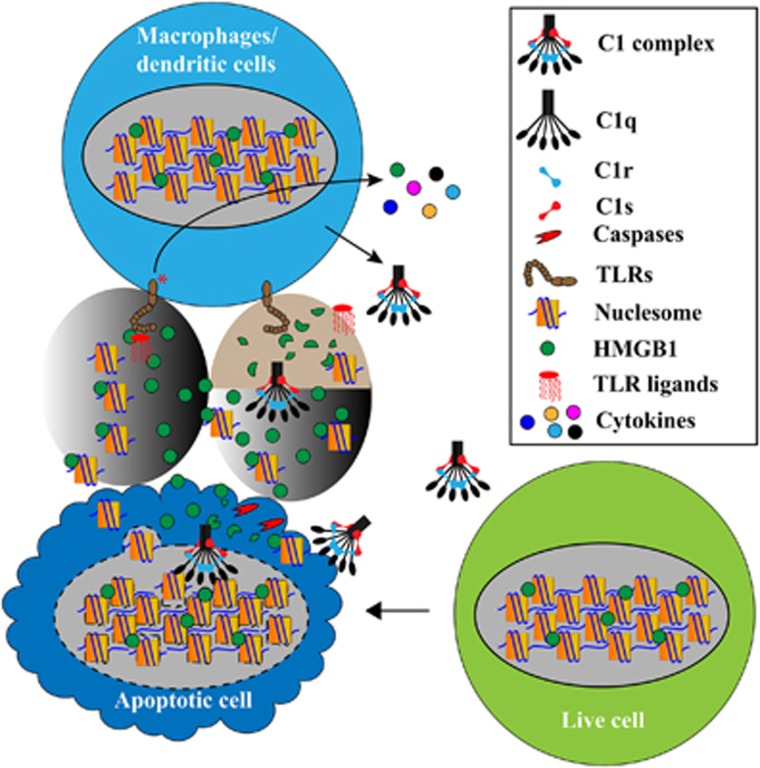
Dead cell trimming to avoid inflammation and autoimmunity. Cells die of different forms between apoptosis and necrosis. Apoptotic cells are often immunosuppressive and non-immunogenic, but necrotic cells leak cellular autoantigens and alarmins/DAMPs and are potentially proinflammatory and immunogenic. Multiple mechanisms exist that normally warrant, besides swift removal of dead cells, the intrinsic demolition of intracellular structures and molecules during normal apoptosis and extrinsic degradation of exposed cellular structures derived from necrotic cells. Using HMGB1 as an example, it is a nuclear protein, which is released when apoptotic cells progress to necrosis. It can be released in complex with chromatin fragments and these complexes have been shown to cause autoantibody production in mice. One of the mechanisms by which HMGB1 activates dendritic cells and macrophages is through its adjuvant effect with LPS. Low levels of LPS or other microbial structures can occur in the circulation. While these subclinical levels of LPS may not significantly activate immune cells through Toll-like receptor 4, the surge of HMGB1 can enable subclinical levels of LPS to activate these cells, leading to inflammation or autoimmunity
